# Study of deaths by suicide of homosexual prisoners in Nazi Sachsenhausen concentration camp

**DOI:** 10.1371/journal.pone.0176007

**Published:** 2017-04-20

**Authors:** Esther Cuerda-Galindo, Francisco López-Muñoz, Matthis Krischel, Astrid Ley

**Affiliations:** 1 Department of Human Anatomy and Embryology, Rey Juan Carlos University, Alcorcón, Madrid, Spain; 2 Faculty of Health Sciences, Camilo José Cela University, Madrid, Spain; 3 Neuropsychopharmacology Unit, Hospital 12 de Octubre Research Institute (i+12), Madrid, Spain; 4 Institute of the History, Philosophy and Ethics of Medicine, Heinrich Heine University Düsseldorf, Düsseldorf, Germany; 5 Memorial and Museum Sachsenhausen, Oranienburg, Germany; Medical University of Vienna, AUSTRIA

## Abstract

Living conditions in Nazi concentration camps were harsh and inhumane, leading many prisoners to commit suicide. Sachsenhausen (Oranienburg, Germany) was a concentration camp that operated from 1936 to 1945. More than 200,000 people were detained there under Nazi rule. This study analyzes deaths classified as suicides by inmates in this camp, classified as homosexuals, both according to the surviving Nazi files. This collective was especially repressed by the Nazi authorities. Data was collected from the archives of Sachsenhausen Memorial and the International Tracing Service in Bad Arolsen. Original death certificates and autopsy reports were reviewed. Until the end of World War II, there are 14 death certificates which state “suicide” as cause of death of prisoners classified as homosexuals, all of them men aged between 23 and 59 years and of various religions and social strata. Based on a population of 1,200 prisoners classified as homosexuals, this allows us to calculate a suicide rate of 1,167/100,000 (over the period of eight years) for this population, a rate 10 times higher than for global inmates (111/100,000). However, our study has several limitations: not all suicides are registered; some murders were covered-up as suicides; most documents were lost during the war or destroyed by the Nazis when leaving the camps and not much data is available from other camps to compare. We conclude that committing suicides in Sachsenhausen was a common practice, although accurate data may be impossible to obtain.

## Introduction

Lesbian, gay, bisexual and transgender populations (LGTB) have been identified as being at high-risk for suicide for over last decades [1, 2]. Recent studies show that individual, social and institutional discrimination against LGTB people may increase risk of mental diseases, substance abuse and suicide [3]. This is collected, in LGTB people, in the Meyer minority stress model, including experiences of prejudice, expectations of rejection, hiding, concealing, internalized homophobia and ameliorative coping processes [4].

National Socialism in Germany was a period during which people were persecuted if they did not conform to social norms. This is particularly true for homosexual men who were strongly repressed [5, 6]. Homosexual women were not usually legally prosecuted, even though in some cases they could be imprisoned as “anti-social individuals” [7]. We can identify discrimination of this population at three different levels: institutional, social and personal.

a) Institutional. From 1871, male homosexuality was illegal in Germany according to article 175 of Penal Code [8]. During the Nazi period, this law remained in effect and the persecution of this group increased. Ideologically, the Nazis believed that homosexuality was a contagious disease and that homosexual persons were a threat not only on the ideal of Aryan race, but on the social policy which needed them as reproductive elements and serve in the armed forces [9]. In the 1930s and 1940s the government presented homosexuality as legally, socially and morally deviant. Numerous films with this view of homosexuality were produced for propaganda purposes [10]. A central Reich Agency to Fight Homosexuality and Abortion was opened at Gestapo headquarters in Berlin in 1936. The first group of men convicted as “homosexuals” had been sent as early as 1933 to Fuhlsbuttel concentration camp for a “re-educational process” [7]. The total number of people classified and imprisoned as “homosexuals” during the Nazi period is estimated between 5,000 and 15,000 [8]. These prisoners were identified in concentration camps with a pink triangle and were treated, together with Jewish prisoners, as the lowest of the groups. They obtained the worst labor assignments, were punished, tortured and often rejected by their fellow prisoners.

Since 1939, ordinances allowed men imprisoned as homosexuals to be released from prisons and concentration camps, if they consented to be castrated. In light of the abuse and high death rates in the camps, it becomes clear that many of the “volunteers” were coerced by the circumstances [11–13]. Homosexuals were also subjected to medical experiments in camps [14], including injections of male hormones in Buchenwald and Neuengamme camps, for testing experimental vaccines in Buchenwald camp [15], or experiments with several psychotropic drugs [16]. It has been confirmed that pink-triangle prisoners had an above average lethality rate: 55% died in the camps, a significantly higher rate than other inmates who were imprisoned for “re-educational proposes” as political prisoners (40%) [17].

b) Social. At the popular level, homosexuals were viewed as “inferior men with animal instincts” [18]. The persecution of homosexual men carried the traditional religious and psychiatric stigmata [19]. The topic of homosexuality as an aberration appeared frequently in the German press, literature and film of the day.

c) Personal. At the individual level, homosexual men continued to be persecuted and isolated. They often had no family support and when they were sent to prisons or concentration camps (*Konzentrationslager*, KZ), families did not visit them and often hid the fact that their relatives were imprisoned as “homosexuals”. Among the prisoners themselves, they were considered morally and socially deviant and nobody wanted to associate with them for fear of also being considered homosexual. These factors made the conditions in the camps especially hard for prisoners detained under Paragraph 175.

Research suggests that the rate of suicides committed by homosexuals is higher than in the population at large and that these rates are increased because of situations of social exclusion [2, 20]. We consider how this hypothesis may translate to an especially dramatic situation of repression in concentration camps, where the incidence of suicides has been described as 10–30 times higher than in the general public in Germany at the time [21, 22]. Anecdotal evidence suggests that the suicide rate among men imprisoned as “homosexuals” in Sachsenhausen was higher than among the general prisoner population of the camp [23]. Perhaps, according to Meyer minority stress model [4], and despite the extreme conditions of the KZ, homosexual prisoners may have experienced additional minority stress, too (i.e., social rejection within inmates), and it might be inferred that suicide rates of homosexual prisoners should be higher.

Sachsenhausen was a concentration camp near Berlin that operated under Nazi rule between 1936 and 1945, mainly for political prisoners. More than 200,000 people were incarcerated there (Sachsenhausen concentration camp web page; available at URL http://www.stiftung-bg.de/gums/en/index.htm). It is estimated that ca. 1,200 prisoners were held as “homosexuals” [24]. Initially, the camp was used to confine political opponents of the Nazi regime, but later also other groups considered as inferior by the Nazis, including Jews, Gypsies and homosexuals [25]. After 1939, more and more citizens of occupied Europe were deported to Sachsenhausen KZ, where they were forced to work as slave laborers in companies of the SS (*Schutzstaffel*) in approximately 100 satellite camps, mostly for military goods factories (see Sachsenhausen concentration camp web page; available at URL http://www.stiftung-bg.de/gums/en/index.htm).

The aim of our study is to analyze historical sources from the archives of Sachsenhausen memorial of suicides committed by prisoners classified as homosexuals. To our knowledge, this is the first time that archival data, including death certificates issued by the Nazi authorities, is analyzed.

## Methods

Material from the archives of Sachsenhausen KZ, where documents concerning the victims imprisoned are conserved, was reviewed. The names and data of all concentration camp prisoners registered as homosexual who committed suicide were examined.

Literature and archives containing data related to suicide and homosexual men were reviewed, including testimonies and trials. Material regarding autopsies performed in case of suicide was studied [26]. Additional files were consulted to reconstruct some biographies at Bundesarchiv (Berlin).

This contribution faces two major methodological challenges: first, archival data remain incomplete, as many documents were systematically destroyed during the “Third Reich” or lost during the war. Second, the data contains different systematic biases. The first bias is one of false negatives: Witness accounts describe some concentration camp prisoners committing suicide by means of stepping into demarcation zones and subsequently being shot or flinging themselves onto electrified fences. None of these cases are registered as suicides; most likely they are filed as prisoners killed trying to escape. The second bias is one of false positives: It is possible that some prisoners were murdered by SS guards or kapos and their deaths filed as suicides. We are aware of these limitations of data and caution the readers against putting too much value on the absolute numbers. Nonetheless we believe that comparative numbers to other concentration camp prisoner groups are valuable.

## Results

From 1936 to the end of the Second World War, 222 suicides are documented in the official files of Sachsenhausen concentration camp (including 17 of them in the pre-war period). Dividing the number of 222 suicides by 200,000 prisoners, we calculate a rate of 111 per 100,000 over the period of eight years. 14 of these total number of 222 registered suicides were of prisoners classified as “175er” (“homosexuals”), which represents 6.3% of registered suicides, and a suicide rate of 1,167 per 100,000 (over the period of eight years) for this population of 1,200 “homosexual” men, 10 times higher than for global inmate population.

From a demographic point of view (Table 1), suicides classified as “homosexuals” were men from 23 and 59 years old (average 45 years old), with a majority of protestant religion (n = 10) and fewer Catholics (n = 3). For one man, undenominated is stated. They were from different social classes and occupational groups (among them one famous artist and one physician). All of them were German citizens, but some were arrested in foreign cities like Prague and Vienna, where the men might have tried to escape persecution. The cause of arrest and imprisonment was the transgression of article 175 of the German Penal Code applicable at that time [7].

**Table 1:** Data on suicide cases committed prisoners classified as homosexuals in Sachsenhausen camp.

Name	Year of birthday	Age	Religion	Profession	Date of imprisonment	Data of suicide	Suicide type	Block
KB	1881	59	Protestant	Baker	23/03/1940	25/05/1940	Hanging	35
DHB	1884	56	Protestant	Salesperson	12/07/1939	03/05/1940	Hanging	14 / 35
AG	1890	49	Protestant	Laborer	18/04/1940	01/06/1940	Hanging	35
PG	1902	40	Catholic	Physician	27/10/1938	08/07/1942	Hanging	14
WH	1905	55	undenominational	Salesperson	27/06/1940	02/07/1940	Hanging	35
PJ	1897	34	Protestant	Laborer	09/06/1940	11/06/1940	Hanging	35
RK	1901	46	Protestant	Waiter	04/05/1940	09/05/1940	Hanging	35
WK	1889	42	Protestant	Bank employee	14/01/1940	05/05/1940	Hanging	35
WM	1913	47	Catholic	Milker	07/05/1940	09/05/1940	Hanging	65 / 35
EKP	1899	32	Protestant	Cook	08/03/1941	17/07/1942	Hanging	14
LR	1885	57	Catholic	Laborer	28/12/1939	14/04/1940	Hanging	35
KS	1903	23	Protestant	Baker	21/03/1940	21/03/1940	Hanging	11
HW	1904	47	Protestant	Salesperson	11/04/1940	16/05/1940	Hanging	35
PW	1894	47	Protestant	Artist	30/05/1940	17/07/1940	Hanging	35

Suicides were recorded between March 1940 and July 1942 and the average time from imprisonment date to suicide was 228 days, although data shows a divergent pattern with one prisoner who committed suicide one day after imprisonment and another inmate who committed suicide after 3 years and 10 months of being in the concentration camp. Five inmates committed suicide in their first internments week, 6 of them before the end of the first year and the other 3 experienced more than one year in the camp. Method for committing suicide was hanging in all the cases. Eleven of the prisoners were incarcerated in Block 35, two in Block 14 and just one in Block 11 Blocks 35 and 11 were part of the "isolation section”. In these separated blocks the men were deprived of contact with the rest of the camp and were also subjected to harsher conditions of detention and constant abuse by especially brutal SS block leaders.

There are 2 autopsy forms from 1940 of corpses that were examined by Dr. Gustav Ortmann, who was the SS camp doctor. He confirmed the cause of death as suicide by hanging and describes the typical external marks of it (Bad Arolsen Archives) [26].

## Discussion

Several studies have revealed a significantly higher rate of suicidal thoughts and actions in homosexually oriented males than in heterosexual males [2, 27]; i.e., they are up to 13.9 times more at risk for a serious suicide attempt, according with Bagley and Tremblay [20]. The mechanisms underlying this increased risk are sufficiently clear [4, 28, 29], and high levels of depression may play an important role [30]. Other important contributory factors are social and personal exclusion. Homosexual men were strongly discriminated against in Nazi Germany and imprisonment in concentration camps was one of the strongest forms of discrimination at the time, including forced labor, torture and incidences of murder. These higher rates of suicidal ideation could become consummated suicides in situations of great vital pressure, as happened with homosexuals interned in Nazi concentration camps [31, 32].

Suicide rates calculated in Sachsenhausen KZ according to our data represent 1.16% of homosexual prisoners: 14 suicides in a calculated population of 1,200 homosexuals imprisoned in Sachsenhausen [24], i.e. a suicide rate of 1,167 per 100,000 (over the period of eight years). This is significantly higher (10 times) than the rate—also calculated from the official files, with the same restrictions—for the general camp population (111 per 100,000). Other authors, such as Lautmann [17], have stated that suicide rates of homosexual inmates were not significantly different from those of the other inmate groups studied in Nazi KZs. The rate we calculated might not be completely accurate, taking into account the methodological challenges discussed above: Examples include prisoners who attempted to escape from the camp, knowing that they would be electrocuted by the electric fence or shot by guards. At the same time, some registered suicides may be murders covered up by the SS guards or might have been committed on orders of the guards or kapos. Nonetheless, we argue that our numbers based on official camp files are comparable with other numbers obtained from official camp files.

Lautmann, using a similar method of calculation [8], estimated the rate of suicides among homosexual prisoners in Buchenwald as 1,000 per 100,000, comparable with our numbers for Sachsenhausen. Some witnesses argue that suicide among prisoners classified as homosexuals in Sachsenhausen was extremely common [23, 33]. Their accounts include methods other than hanging, which were not classified as suicide in the official files.

The higher number of man imprisoned as “homosexuals” can be explained by the especially hard repression that they suffered in Sachsenhausen KZ. Working conditions for homosexual inmates, who worked at the satellite camp at the brick works (*Klinkerwerk*) are described as even worse than in the main camp. Reports include repeated shootings (more than 200 in total) of forced laborers who were flung beyond camp borders and then shot for attempting to escape [23], as well as housing in specific isolation units. This might indicate that even under generally inhumane conditions, a mistreatment of some prisoners beyond the norm of the concentration camp significantly increased the suicide rate in this population.

Prisoners who committed suicide were of all age range and from different social classes and occupations. No official statistics about the age of people who committed suicide in Nazi concentration camps are reported, but in our study we found that the majority was more than 40 years old (78.5%). Compared to the whole inmate population, they are quite old, as the average age, especially of the Polish and Czech prisoners was much lower. But compared to the group of the German inmates (which they were) they match the age.

Among them was one famous singer and cabaret artist named Paul O’Montis, pseudonym of Paul Wendel (Budapest, April 3, 1894—Sachsenhausen KZ, July 17, 1940), who was persecuted by the Nazis. After the annexation of Austria in 1938, he fled to Prague. Montis was arrested in 1939 and deported first to Zagreb and later to Lodz. He was deported to Sachsenhausen in late May 1940 and died six weeks later at the age of 46 years. Even when his death was reported as suicide, witnesses stated that this was a covered-up of a murder by the kapo of the block he was interned. Another suicidee was Paul Grundmann (Berlin, April 24, 1902 –Sachsenhausen KZ, July 8, 1942), who had studied medicine from 1921 to 1933. After receiving his MD, he practiced medicine in Berlin. He was sent to Brual/Rhede (Ems) internment camp in the North-west of Germany (during or after November 1938), was stripped of his license to practice medicine by the Nazis in July of 1939 and sent to Sachsenhausen KZ in November 1941. A third prisoner who is stated to have committed suicide was Emil Pfensig who was assigned to the cement plant. The commander Rudolf Hess was convinced that sexual orientation could be changed through hard labor [34]. The homosexual prisoners labored quarrying clay and making bricks in the camp [10]: two-thirds of them died within two months [35]. LD von Classen-Neudegg [36], Leo Clasen pseudonym, describes the death of some 300 homosexuals laboring in the cement plant. Under these extremely hard conditions, 9 inmates committed suicide based in testimonies [23]. Emil Pfensig was one of them, the names of the others are not known.

The data shows that the majority of suicides were committed in the first years of imprisonments, which is in agreement with other authors [37]. Almost 30% committed suicide during the first days, which is in agreement with those authors who attribute this high rate to initial shock of imprisonment [38]. However, in our sample, the average time of internment until the commission of suicide was 7.5 months. Especially in the period after 1939, such a long period of imprisonment in a concentration camp might eliminate most of the survival protective factors, such as fear of suicide or social reprobation, moral or religious values [39, 40]. In the case of homosexual inmates, some of these protective factors, as familiar responsibility or presence of relatives, did not exist [41, 42]. Other factors, such as separation from family, suspicion of death by the relatives, physical suffering, illness, hopelessness or certainty of extermination could also contribute to eliminate survival capacity [43, 44]. In these harsh circumstances of repression, suicide could be perceived by some prisoners as the last way of escape from unbearable conditions [45].

The first homosexual prisoners deported to Sachsenhausen were hosted with other prisoner categories in all the blocks [46]. Homosexual prisoners were forced to sleep in nightshirts and hold their hands outside the covers to prevent masturbation [36]. From the late summer of 1939, the prisoners marked as "homosexual" with pink triangles were housed together in the "isolation section", among others: Block 35. This is in agreement with the data according to which most of the prisoners were housed in Block 35 when they committed suicide. They were isolated there to avoid “homosexuality propagation” and they were separated from the rest of the camp by a wall (Sachsenhausen concentration camp web page; available at URL http://www.stiftung-bg.de/gums/en/index.htm). One inmate committed suicide when he was moved to the punishment block (Block 11): the conditions in this block were extremely hard [47], so that this suicide can be explained by fear or consequence of torture reported by some authors [37, 48]. From our data, we cannot conclude that suicides could have seasonal variation or possible contagion effects, including suicide of celebrities (see O'Montis), as some authors have recently found [49].

We could differentiate two forms of behavior when committing suicide in Nazi concentration camps; impulsive behavior (i.e., crossing SS guard lines to get shot or to touch electrified barbed wire fences) and premeditated suicide (by hanging or through poison) [50–52]. The method of suicide in all cases in our sample was by hanging, most likely because the other forms of suicide were not filed as such. These methods require more reflexion, and require isolated places [52, 53]. Some authors asses that the most common way of suicide was flinging oneself onto the wires [37], but they are based on witness reports and the testimonies could be influenced by the fact that death by flinging onto the electrified fence is more shocking than death by hanging in which the inmate commits suicide in a lonely place. Another important factor is that inmates killed on the fences or shot by guards would be classified as “killed in an escape attempt” instead of suicide. Our results are in agreement with authors [54] who asses that prisoners used methods available to them; guns and knives were banned in the camp for inmates, of course. Most of the suicides were committed during night hours, when vigilance was lower; eight suicides of our sample were made between 2:00 and 6:30 hours. As mentioned above, it remains unclear if or how many of the suicides were ordered by guards or kapos [52, 55].

After beginning of the war in 1939, SS guards could in practice kill prisoners without trial; they kept covering-up some murders as suicides [54]. In some cases of famous inmates they preferred to cover-up the murder avoiding one scandal. It could be the case of the famous international singer Paul O’Montis. There are some reports of witness who relate how they could differentiate when one prisoner had committed suicide or he had been killed by strangulation, observing the marks in the prisoner’s neck when the corpses were stored in the infirmary before cremating [23].

## Conclusions

In this paper, we present the first study of homosexual suicides in Sachsenhausen KZ. Camp experience for homosexual prisoners was especially harrowing, because of the special repression suffered by this group. We have found a higher number of suicides in this camp when compared to the general prisoner population at Sachsenhausen under Nazi control and under Soviet rule [21]. The number is also higher than available rates of suicides of prisoners classified as homosexuals at Buchenwald, where the conditions were comparably less atrocious. However, our study has several limitations: not all suicides are registered; some murders were covered-up as suicides; most documents were lost during the war or destroyed by the Nazis when leaving the camps and not much data is available from other camps to compare. Nonetheless, our study contributes to an important field of research in which much work remains to be done. We conclude that higher rate of suicides among homosexual inmates can be correlated with the higher degree of repression against this group in Sachsenhausen KZ.
